# Supplementary material for: UBE2J2 sensitizes the ERAD ubiquitination cascade to changes in membrane lipid saturation
Source: Nat Commun. 2025 Oct 9;16:8973. doi: 10.1038/s41467-025-64777-1 (PMC12511567; doi:10.1038/s41467-025-64777-1)
Supplement: Supplementary file 1 — Supplementary Information [file 41467_2025_64777_MOESM1_ESM.pdf]

SUPPLEMENTARY INFORMATION

for

UBE2J2 sensitizes the ERAD ubiquitination cascade to changes in membrane lipid saturation

Aikaterini Vrentzou[1], Florian Leidner[2], Claudia C. Schmidt[1,3], Helmut Grubmüller[2], Alexander Stein[1]

1 Research Group Membrane Protein Biochemistry, Max Planck Institute for Multidisciplinary Sciences, Am Fassberg 11, D-37077 Göttingen, Germany

2 Department of Theoretical and Computational Biophysics, Max Planck Institute for Multidisciplinary Sciences, Am Fassberg 11, D-37077 Göttingen, Germany

3 Current Address: ETH Zürich, Otto-Stern-Weg 3, 8093 Zürich, Switzerland

Correspondence to alexander.stein@mpinat.mpg.de

The PDF file includes:

- Supplementary Figures 1-10
- Supplementary Tables 1-5
- Supplementary Discussion
- Supplementary References

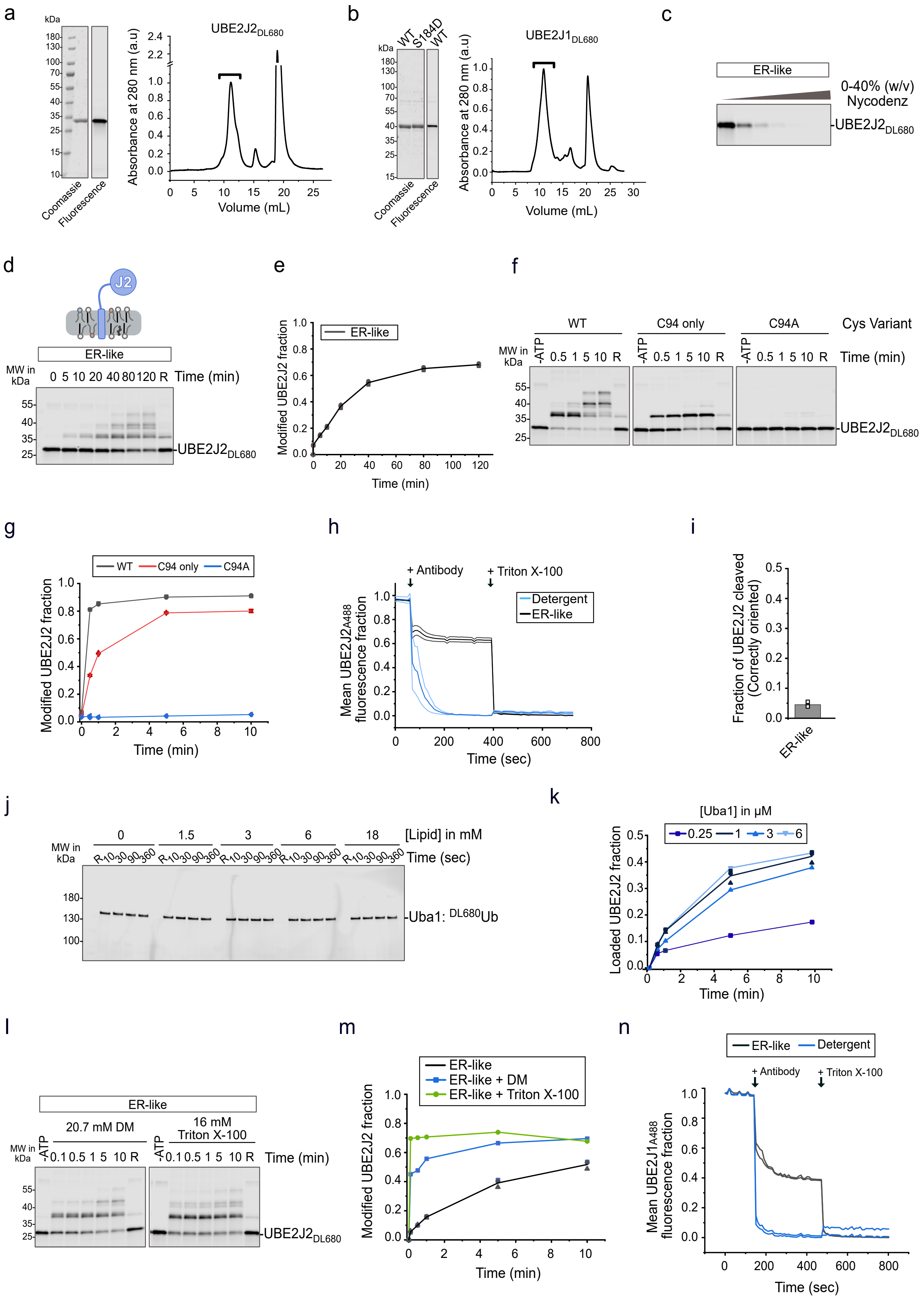
a
kDa
180
130
100
70
55
40
35
25
15
10
Coomassie
Fluorescence
UBE2J2DL680
Absorbance at 280 nm (a.u)
Volume (mL)
b
WT
S184D
WT
UBE2J1DL680
c
ER-like
0-40% (w/v) Nycodenz
UBE2J2DL680
d
J2
ER-like
MW in kDa
0 5 10 20 40 80 120 R
Time (min)
UBE2J2DL680
e
ER-like
Modified UBE2J2 fraction
Time (min)
f
WT
C94 only
C94A
Cys Variant
-ATP
0.5 1 5 10 R
Time (min)
UBE2J2DL680
g
WT
C94 only
C94A
Modified UBE2J2 fraction
Time (min)
h
+ Antibody
+ Triton X-100
Detergent
ER-like
Mean UBE2J2A488 fluorescence fraction
Time (sec)
i
Fraction of UBE2J2 cleaved (Correctly oriented)
ER-like
j
0
1.5
3
6
18
[Lipid] in mM
R 10 30 90 360
Time (sec)
Uba1: DL680Ub
k
[Uba1] in μM
0.25
1
3
6
Loaded UBE2J2 fraction
Time (min)
l
ER-like
20.7 mM DM
16 mM Triton X-100
-ATP
0.1 0.5 1 5 10 R
Time (min)
UBE2J2DL680
m
ER-like
ER-like + DM
ER-like + Triton X-100
Modified UBE2J2 fraction
Time (min)
n
ER-like
Detergent
+ Antibody
+ Triton X-100
Mean UBE2J1A488 fluorescence fraction
Time (sec)

Supplementary Figure 1. Purification and characterization of UBE2J1/2 and assay controls (related to Fig. 1). a,b Purified, fluorescently labelled UBE2J2 **(a)** and UBE2J1 **(b)** were analyzed by SDS-PAGE and visualized by Coomassie staining or fluorescence scanning. Size exclusion chromatography performed on a Superdex 200 10/300 GL column, shows major elution peaks for UBE2J2 and UBE2J1 (brackets) and later peaks for free fluorescent peptide. **c** Flotation assay on a 0-40% (w/v) Nycodenz gradient assessing UBE2J2 reconstitution in liposomes of ER-like composition. The fractionated gradient analyzed by SDS-PAGE and fluorescence scanning demonstrates UBE2J2 migrates with liposomes to top fractions. **d** Extended time course of ubiquitin loading and auto-ubiquitination of UBE2J2$_{DL680}$ reconstituted in ER-like liposomes (P/L= 1: 32,000). **e** Quantification of **(d)**, n=3. **f** Ubiquitin loading of WT, C94 only (C33S/C88S/C157S) or C94A UBE2J2$_{DL680}$ in 0.03% (w/v) DDM. **g** Quantification of **(f)**, n=2. **h** Analysis of UBE2J2 orientation in ER-like liposomes (P/L= 1:32,000) assessed by antibody-mediated quenching of AlexaFluor 488 fluorescence. Correctly-oriented UBE2J2$_{A488}$ is protected from antibody-mediated quenching (64 ± 2 % at t = 200 s); solubilization with 1% Triton X-100 allows quenching. Reactions in 0.03% (w/v) DDM serve as a positive control (n=3 for detergent, n=2 for ER-like liposomes, including 2 technical replicates for each). Bands represent the mean and error from all replicates. **i** Analysis of UBE2J2 orientation in ER-like liposomes (P/L= 1:32,000) by protease protection. Quantification of Ulp1-mediated cleavage of His$_{14}$-SUMO-UBE2J2$_{DL680}$ reconstituted in ER-like liposomes indicates 95% correct orientation. Ulp1 cleaves the N-terminal SUMO tag of correctly-oriented UBE2J2 (n=3). **j** Loading of the E1 Uba1 with fluorescently labeled ubiquitin in the presence of protein-free ER-like liposomes at indicated lipid concentrations; analyzed by non-reducing SDS-PAGE and fluorescence scanning. **k** Effect of increasing E1 concentration on ubiquitin loading of UBE2J2$_{DL680}$ in ER-like liposomes; analyzed by non-reducing SDS-PAGE and fluorescence scanning. **l** Ubiquitin loading of UBE2J2$_{DL680}$ in detergent-solubilized liposomes. UBE2J2$_{DL680}$ was reconstituted in ER-like liposomes (P/L= 1:32,000). Prior to ubiquitin loading, liposomes were solubilized with n-decyl-β-D-maltoside (DM) or Triton X-100 at the indicated concentrations; analyzed by non-reducing SDS-PAGE and fluorescence scanning. **m** Quantification of **(l)**. Graph contains data from Fig. 1c for comparison. **n** As in **(h)**, for UBE2J1$_{A488}$. R=reduced. a.u. = arbitrary units. ER-like liposomes contained 60 mol% POPC, 20 mol% DOPE, 10 mol% DOPS, 10 mol% cholesterol.

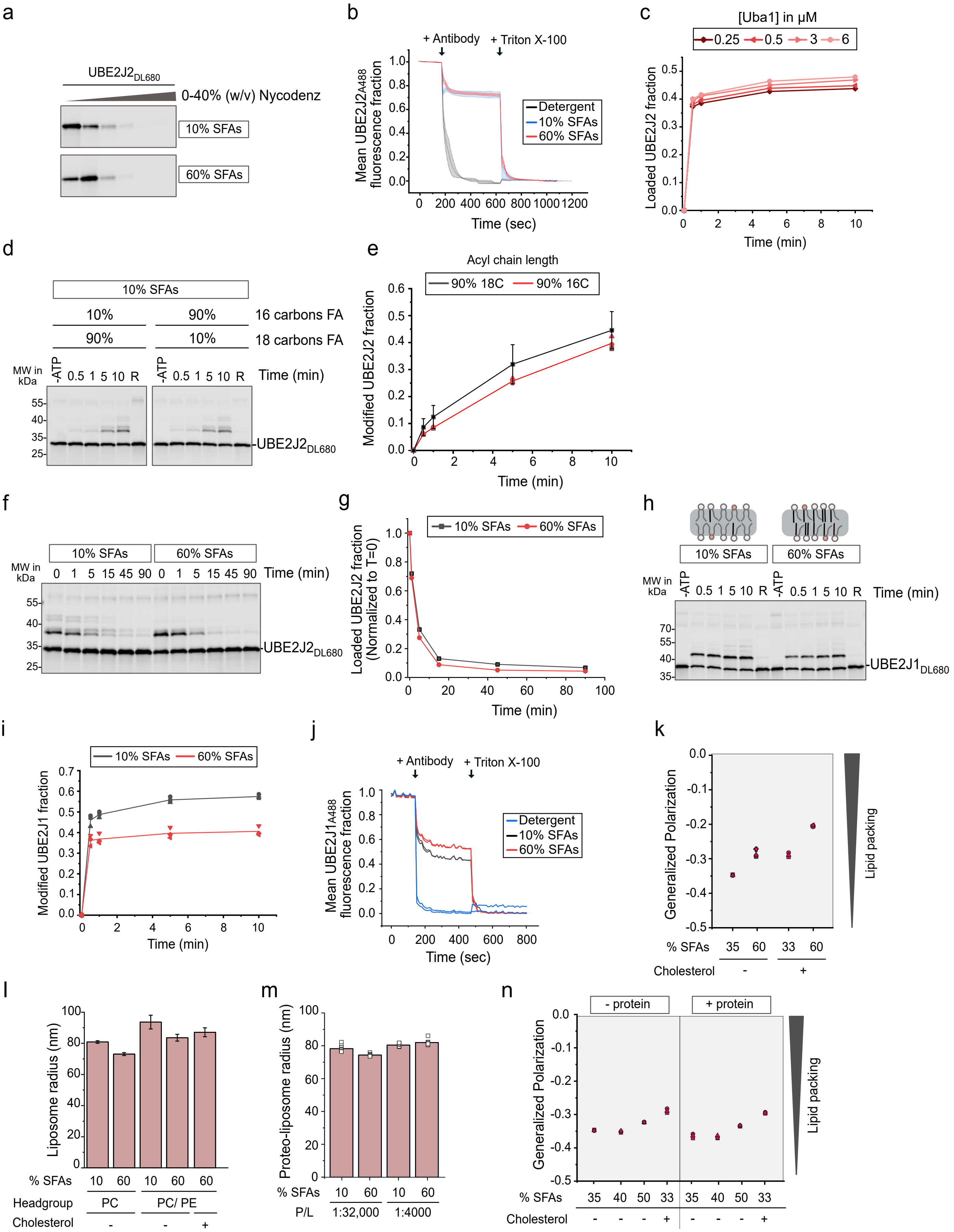

a
UBE2J2DL680
0-40% (w/v) Nycodenz
10% SFAs
60% SFAs
b
+ Antibody
+ Triton X-100
Mean UBE2J2A488 fluorescence fraction
Detergent
10% SFAs
60% SFAs
Time (sec)
c
[Uba1] in µM
0.25
0.5
3
6
Loaded UBE2J2 fraction
Time (min)
d
10% SFAs
10%
90%
16 carbons FA
90%
10%
18 carbons FA
MW in kDa
-ATP 0.5 1 5 10 R
Time (min)
UBE2J2DL680
e
Acyl chain length
90% 18C
90% 16C
Modified UBE2J2 fraction
Time (min)
f
10% SFAs
60% SFAs
MW in kDa
0 1 5 15 45 90
Time (min)
UBE2J2DL680
g
10% SFAs
60% SFAs
Loaded UBE2J2 fraction (Normalized to T=0)
Time (min)
h
10% SFAs
60% SFAs
MW in kDa
-ATP 0.5 1 5 10 R
Time (min)
UBE2J1DL680
i
10% SFAs
60% SFAs
Modified UBE2J1 fraction
Time (min)
j
+ Antibody
+ Triton X-100
Mean UBE2J1A488 fluorescence fraction
Detergent
10% SFAs
60% SFAs
Time (sec)
k
Generalized Polarization
Lipid packing
% SFAs 35 60 33 60
Cholesterol - +
l
Liposome radius (nm)
% SFAs 10 60 10 60 60
Headgroup PC PC/ PE
Cholesterol - - +
m
Proteo-liposome radius (nm)
% SFAs 10 60 10 60
P/L 1:32,000 1:4000
n
- protein
+ protein
Generalized Polarization
Lipid packing
% SFAs 35 40 50 33 35 40 50 33
Cholesterol - - - + - - - +

Supplementary Figure 2. UBE2J2/1 reconstitution and activity in liposomes of defined acyl chain composition (related to Fig. 2). a Flotation assay to assess UBE2J2 reconstitution in liposomes with lipid compositions as in Fig. 2. UBE2J2 migrates with liposomes to the top fractions of a 0-40% (w/v) Nycodenz density gradient; gradient fractions analyzed by SDS-PAGE and fluorescence scanning. **b** Orientation analysis of UBE2J2 in liposomes with 10% or 60% SFAs (P/L= 1: 32,000), assessed by antibody-mediated quenching of AlexaFluor 488 fluorescence. Correct orientation protects from quenching; solubilization with 1% Triton X-100 leads to quenching of also correctly oriented UBE2J2$_{A488}$. Antibody-mediated quenching of UBE2J2$_{A488}$ fluorescence in 0.03% (w/v) DDM solution was used as a positive control (n=2 for each, including technical replicates for each). Bands represent the error and mean of technical replicates. **c** Effect of E1 concentration on ubiquitin loading of UBE2J2$_{DL680}$ reconstituted in liposomes with 50% SFA content; analyzed by non-reducing SDS-PAGE and fluorescence scanning. **d** Ubiquitin loading of UBE2J2$_{DL680}$ in liposomes containing 10% SFA with indicated acyl chain lengths (P/L= 1:32,000). In comparison to the standard 10% SFA liposomes, di-oleyl-PC (18:1) was replaced with 1,2-dipalmitoleoyl-PC (16:1). **e** Quantification of **(d)**, n=3. Graph contains data from Fig. 2b for comparison. **f** Discharge kinetics of ubiquitin-loaded UBE2J2, reconstituted in liposomes with 10% or 60% SFAs (P/L=1:32,000). UBE2J2$_{DL680}$ was loaded (10 min for 10% SFAs, 1 min for 60% SFAs, 50 nM UBE2J2, 20 nM E1), diluted 10-fold, and discharge monitored in quench buffer with EDTA and 2 µM Rad6 to minimize residual E1 activity. **g** Quantification of the loaded UBE2J2 fraction in **(f)**. Data were normalized to timepoint 0. **h** Ubiquitin loading of UBE2J1$_{DL680}$, in liposomes with the indicated SFA content. **i** Quantification of reactions as in **(h)** (n=2). **j** As in **(b)**, for UBE2J1 (n=2 technical replicates). **k** The effect of cholesterol on the lipid packing in liposomes assessed by C-Laurdan fluorescence; emission spectra were recorded at 37°C and generalized polarization calculated as described[1] (n≥3). **l, m** Dynamic light scattering analysis of protein-free **(l)** and UBE2J2-containing **(m)** liposomes with indicated lipid compositions and protein/lipid ratios. Bars represent mean ± s.d. or triplicates **(l)**, or the mean of multiple technical replicates. **n** The effect of proteins on the lipid packing of liposomes with variable lipid acyl chain saturation and cholesterol content assessed with C-Laurdan fluorescence as in **(k)**, but comparing protein-free with UBE2J2 liposomes (n≥3). Unless indicated otherwise, E2s were reconstituted at a P/L of 1:32,000. R = reduced. Detailed liposome compositions are given in Table 1.

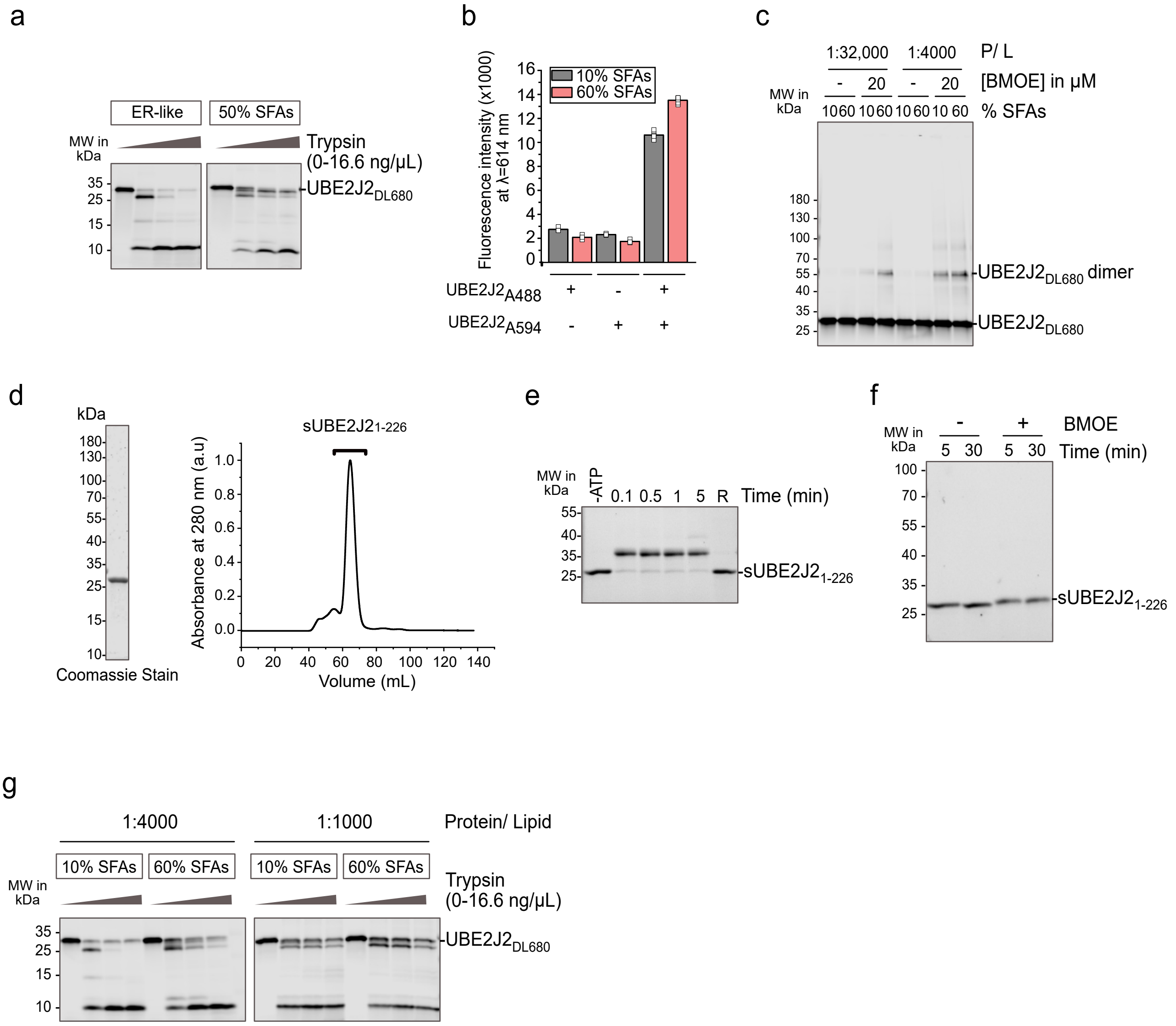
a
ER-like
50% SFAs
MW in kDa
Trypsin (0-16.6 ng/µL)
UBE2J2DL680
b
Fluorescence intensity (x1000) at λ=614 nm
10% SFAs
60% SFAs
UBE2J2A488
UBE2J2A594
c
1:32,000
1:4000
P/ L
[BMOE] in µM
% SFAs
UBE2J2DL680 dimer
UBE2J2DL680
d
kDa
Coomassie Stain
sUBE2J21-226
Absorbance at 280 nm (a.u)
Volume (mL)
e
-ATP
Time (min)
sUBE2J21-226
f
BMOE
Time (min)
sUBE2J21-226
g
1:4000
1:1000
Protein/ Lipid
10% SFAs
60% SFAs
Trypsin (0-16.6 ng/µL)
UBE2J2DL680

Supplementary Figure 3. UBE2J2 reconstitution, structural analysis, and assay (related to Fig. 3). a Limited proteolysis of UBE2J2$_{DL680}$ reconstituted in liposomes with indicated SFA content (P/L = 1:32,000). Liposomes were treated with increasing trypsin concentrations (0, 1.9, 5.6 or 16.6 ng/µl) at 25 °C for 30 minutes. Samples were analyzed by SDS-PAGE and fluorescence scanning. **b** Specificity of acceptor emission in FRET experiments (see Fig. 3c, d). Liposomes were reconstituted with UBE2J2$_{A488}$ (donor), UBE2J2$_{A594}$ (acceptor), or both (P/L = 1:20,000 and 1:5000 for UBE2J2$_{A488}$ and UBE2J2$_{A594}$, respectively; emission at 614 nm (excitation 480 nm) was measured at 25 °C. Data represent two independent reconstitutions, each measured in triplicate (n=2). **c** Cysteine crosslinking of UBE2J2$_{DL680}$ in liposomes (P/L = 1:32,000 or 1:4000) using 20 µM BMOE for 30 min at 25°C. Products analyzed by SDS–PAGE and fluorescence scanning. **d** Purified soluble UBE2J2 (sUBE2J2$_{1\text{-}226}$) analyzed by SDS-PAGE and Coomassie staining. SEC of sUBE2J2$_{1\text{-}226}$ was performed with a HiLoad S75 16/60 column. Brackets indicate the elution peak of soluble UBE2J2. **e** Ubiquitin loading of sUBE2J2$_{1\text{-}226}$ analyzed by SDS-PAGE and stain-free scanning. R= reduced with β-mercaptoethanol. **f** Crosslinking of sUBE2J2$_{1\text{-}226}$ with 2 mM BMOE for 30 min at 25°C, analyzed by SDS–PAGE and stain-free scanning. **g** Limited proteolysis of UBE2J2$_{DL680}$ reconstituted in liposomes with indicated SFA content as in **(a)**, but with P/L = 1:4000 or 1:1000. Detailed liposome compositions are given in Table 1.

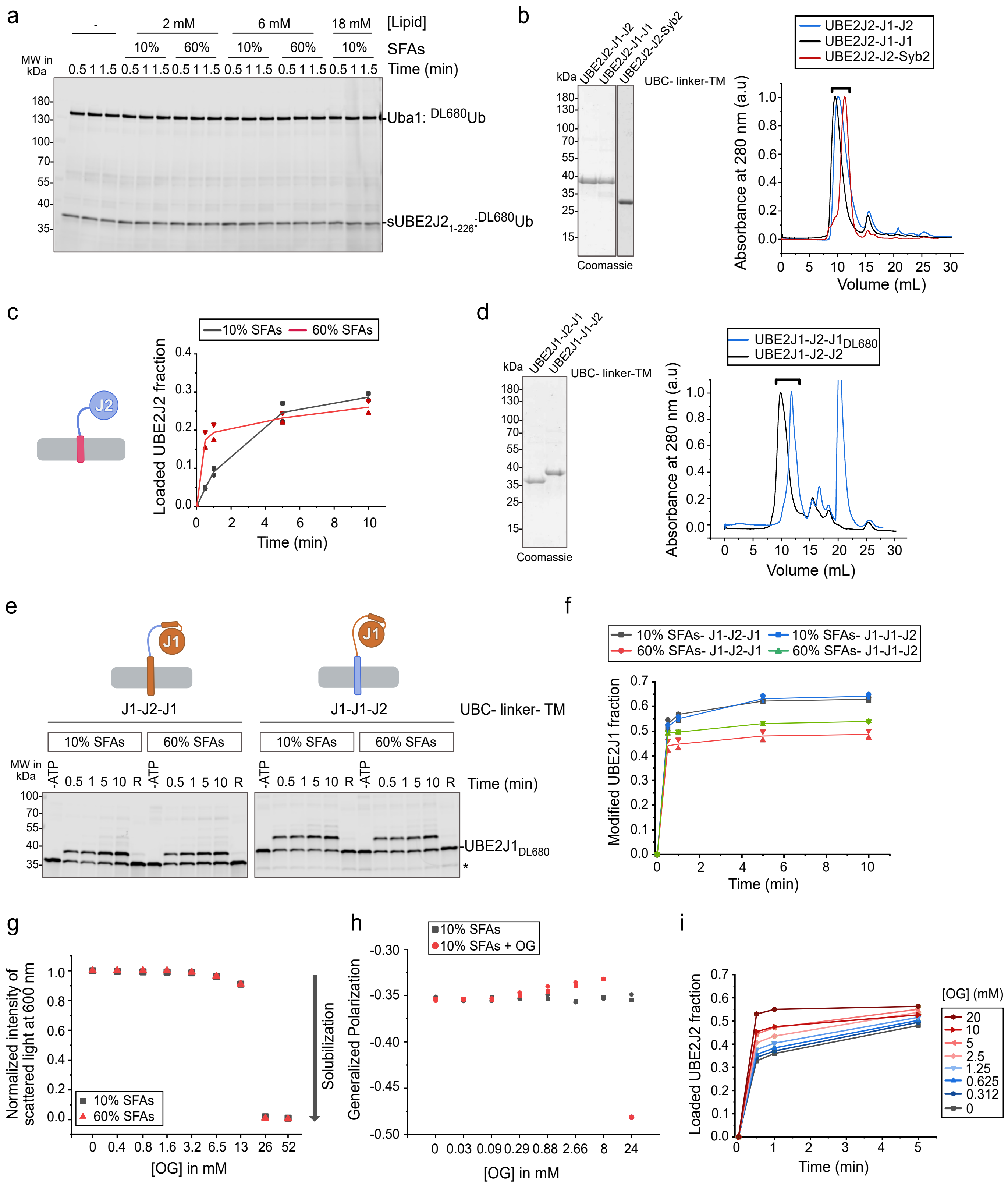

a
[Lipid]
SFAs
Time (min)
Uba1: DL680Ub
sUBE2J2 1-226: DL680Ub
b
UBC- linker-TM
Coomassie
UBE2J2-J1-J2
UBE2J2-J1-J1
UBE2J2-J2-Syb2
Absorbance at 280 nm (a.u)
Volume (mL)
c
10% SFAs
60% SFAs
Loaded UBE2J2 fraction
Time (min)
d
UBE2J1-J2-J1
UBE2J1-J1-J2
UBE2J1-J2-J2
e
J1-J2-J1
J1-J1-J2
UBC- linker- TM
Time (min)
UBE2J1 DL680
f
10% SFAs- J1-J2-J1
10% SFAs- J1-J1-J2
60% SFAs- J1-J2-J1
60% SFAs- J1-J1-J2
Modified UBE2J1 fraction
g
Normalized intensity of scattered light at 600 nm
Solubilization
[OG] in mM
h
10% SFAs + OG
Generalized Polarization
i
[OG] (mM)
Loaded UBE2J2 fraction

Supplementary Figure 4. Characterization of UBE2J2/1 chimeras and sensitivity to membrane composition (related to Fig. 4). a Loading of sUBE2J2$_{1\text{-}226}$ with fluorescent ubiquitin in the presence of protein-free liposomes. **b** Purified, fluorescently labelled UBE2J2 chimeras with the disordered linker or the transmembrane segment (TMS) of UBE2J1 or the TMS of Syb2, analyzed by SDS-PAGE and Coomassie staining. Size-exclusion chromatography (SEC, Superdex 200 10/300 GL) shows elution peaks for chimeras (brackets). **c** Quantification of ubiquitin loading of UBE2J2 with the TMS of Syb2, reconstituted in PC/PE liposomes with the indicated saturated fatty acid (SFA) content (n=2). **d** Purified, fluorescently labelled UBE2J1 chimeras with the disordered linker or the TMS of UBE2J2, analyzed by SDS-PAGE and Coomassie staining or fluorescence scanning. SEC (Superdex 200 10/300 GL) shows elution peaks for chimeras (brackets); late peak indicates free fluorescent peptide. **e** Ubiquitin loading of UBE2J1 chimeras bearing the TMS or disordered linker of UBE2J2, reconstituted in PC/PE liposomes with the indicated SFA content. Asterisk indicates a contaminating protein. **f** Quantification of reactions in **(e)** (n=2). **g** Solubilization of protein-free liposomes upon addition of increasing *n*-octyl-β-D-glucoside (OG) concentrations measured by loss of light scattering. **h** Effect of OG on lipid packing in liposomes with 10% SFA content assessed with C-Laurdan fluorescence; emission spectra recorded at 25 °C, before (black) and after (red) the OG addition (n=2). Generalized polarization calculated as described[1]. **i** Ubiquitin loading of UBE2J2$_{DL680}$ reconstituted in liposomes (60% SFAs, P/L= 1;32,000) after incubation with the indicated OG concentrations for 10 min. Detailed liposome compositions are given in Table 1.

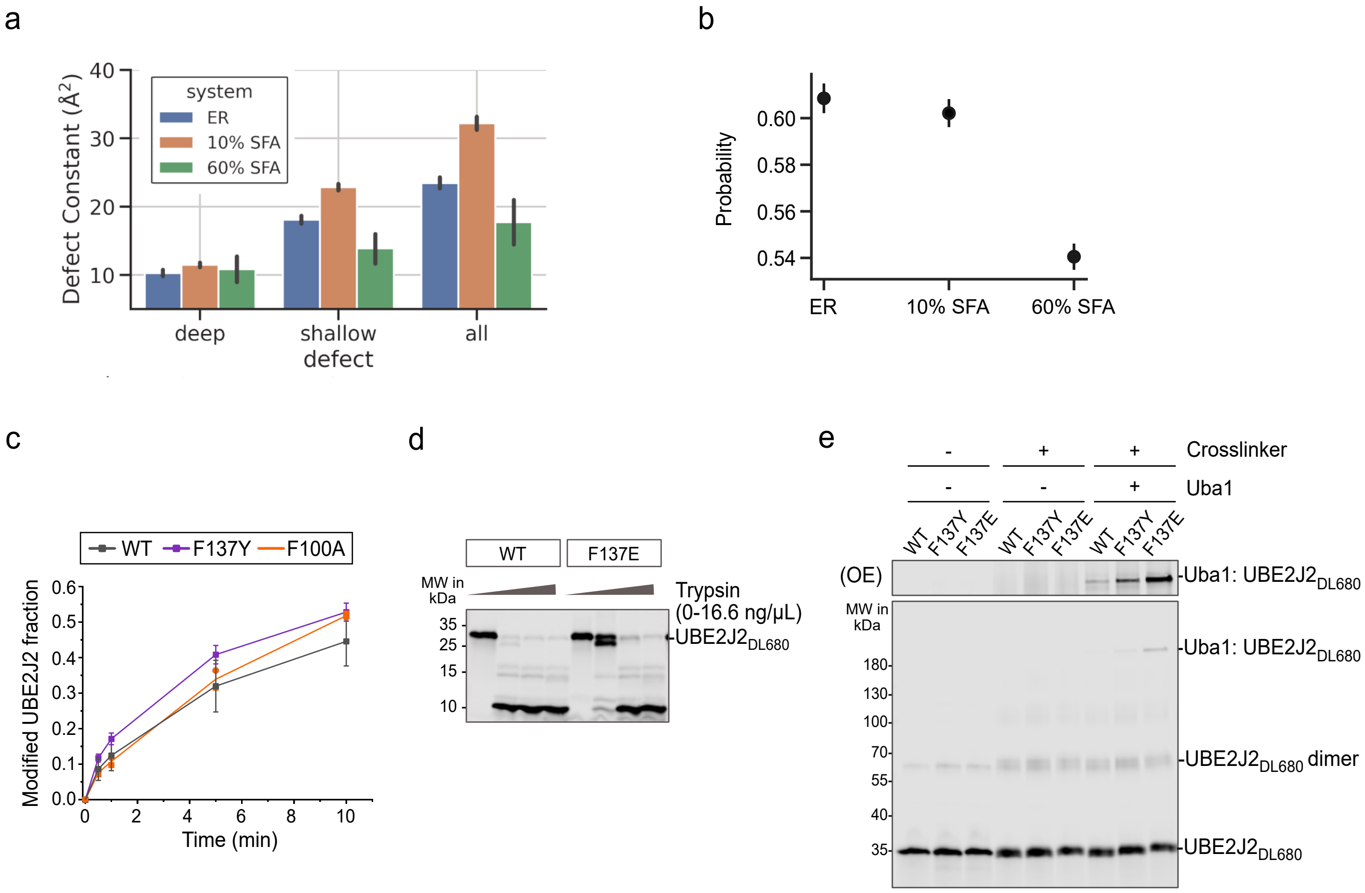

a
b
c
d
e
system
ER
10% SFA
60% SFA
Defect Constant (Å^2)
deep
shallow
defect
all
Probability
ER
10% SFA
60% SFA
WT
F137Y
F100A
Modified UBE2J2 fraction
Time (min)
WT
F137E
MW in kDa
Trypsin (0-16.6 ng/μL)
UBE2J2$_{DL680}$
Crosslinker
Uba1
(OE)
Uba1: UBE2J2$_{DL680}$
UBE2J2$_{DL680}$ dimer

Supplementary Figure 5. Molecular dynamics simulations and characterization of the F137E mutant (related to Fig. 4). a Bar graph of membrane packing defect sizes for different lipid compositions. The packing defect constant (π) relates to the probability p(A) of finding a lipid packing defect in an area A, described by $p(A) = b \times e^{-\frac{A}{\pi}}$. The packing defect constant represents the probability of encountering larger packing defects. PackMem was used to calculate and group membrane defects[2]. **b** Probability of UBE2J2 adopting one of the membrane-bound conformations in membranes of different lipid compositions, as determined by molecular dynamics simulations. Error bars show the 95% confidence intervals calculated from bootstrap resampling (n_resample = 10000). **c** Ubiquitin loading of WT, F137Y or F100A $UBE2J2_{DL680}$, reconstituted in liposomes with 10% SFA content (P/L= 1:32,000; n=7 for WT, n=3 for F137Y, n=2 for F100A). **d** Limited proteolysis assay on WT or F137E $UBE2J2_{DL680}$ reconstituted in liposomes with indicated SFA content (P/L = 1:32,000) using increasing trypsin concentrations (0, 1.9, 5.6 or 16.6 ng/µl) at 25 °C for 30 minutes; analyzed by SDS-PAGE and fluorescence scanning. **e** Cysteine crosslinking to assess interaction of UBE2J2 (WT, F137Y, or F137E) with E1 in liposomes with the 10% SFA content, in the presence or absence of 1,2-bismaleimidoethane (BMOE). Samples collected after 10 minutes, analyzed by SDS-PAGE and fluorescence scanning (OE= overexposed). UBE2J2 was reconstituted at P/L = 1:32,000. Detailed liposome compositions are given in Table 1.

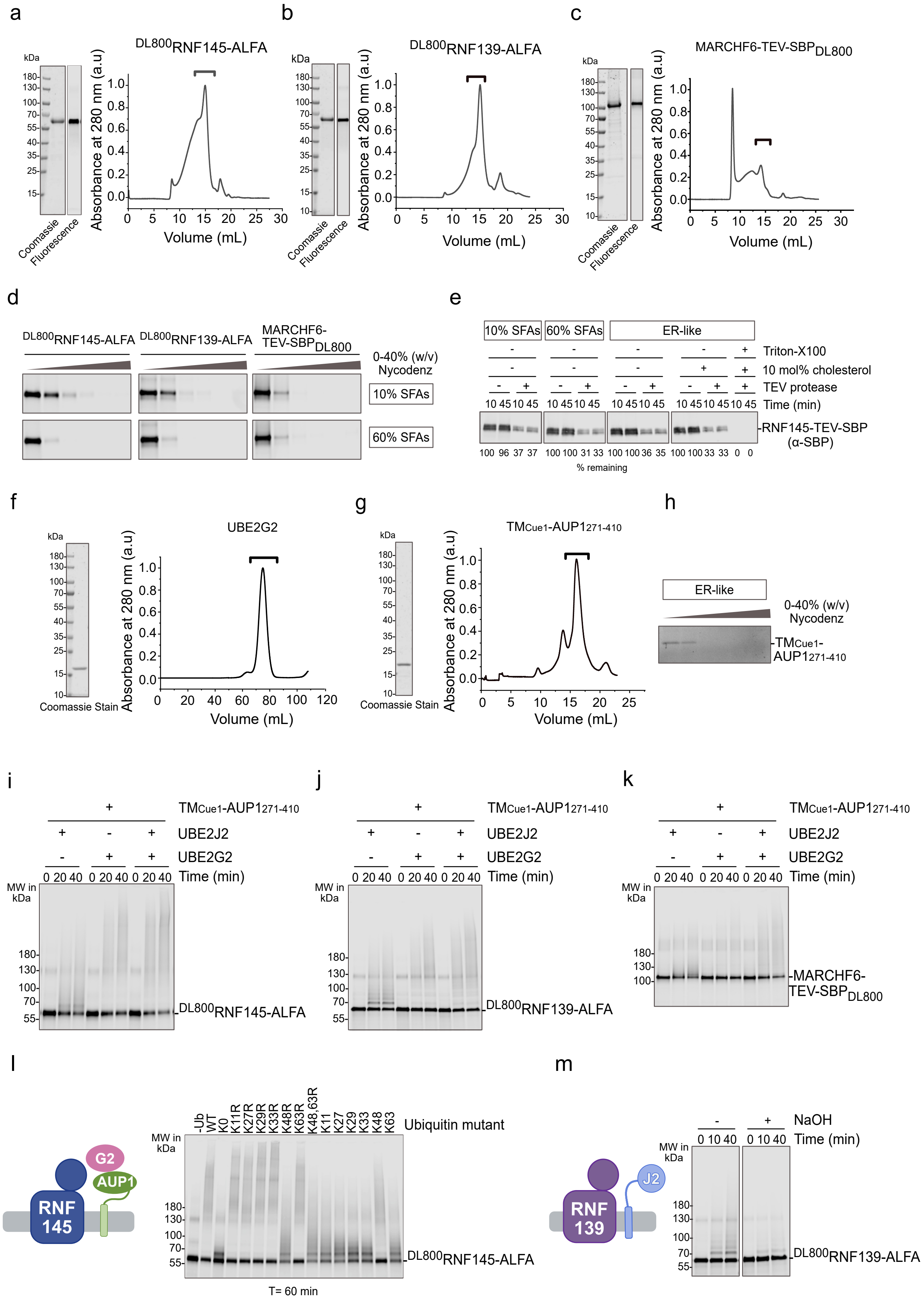
a
DL800RNF145-ALFA
b
DL800RNF139-ALFA
c
MARCHF6-TEV-SBPDL800
kDa
Coomassie
Fluorescence
Absorbance at 280 nm (a.u)
Volume (mL)
d
DL800RNF145-ALFA
DL800RNF139-ALFA
MARCHF6-TEV-SBPDL800
0-40% (w/v) Nycodenz
10% SFAs
60% SFAs
e
10% SFAs
60% SFAs
ER-like
Triton-X100
10 mol% cholesterol
TEV protease
Time (min)
RNF145-TEV-SBP (α-SBP)
100 96 37 37 100 100 31 33 100 100 36 35 100 100 33 33 0 0
% remaining
f
UBE2G2
Coomassie Stain
g
TMCue1-AUP1271-410
h
ER-like
0-40% (w/v) Nycodenz
TMCue1-AUP1271-410
i
j
k
TMCue1-AUP1271-410
UBE2J2
UBE2G2
Time (min)
MW in kDa
DL800RNF145-ALFA
DL800RNF139-ALFA
MARCHF6-TEV-SBPDL800
l
G2
AUP1
RNF 145
-Ub WT K0 K11R K27R K29R K33R K48R K63R K48,63R K11 K27 K29 K33 K48 K63
Ubiquitin mutant
DL800RNF145-ALFA
T= 60 min
m
J2
RNF 139
NaOH
Time (min)
DL800RNF139-ALFA

Supplementary Figure 6. Characterization and activity of E3 ubiquitin ligases and partners in reconstituted systems (related to Fig. 5). a-c Purified, fluorescently labelled E3 ubiquitin ligases RNF145-ALFA **(a)**, RNF139-ALFA **(b)**, and MARCH6-TEV-SBP **(b)**, analyzed by SDS-PAGE with Coomassie staining or fluorescence scanning. Size-exclusion chromatography (Superose 6 10/300 GL) shows elution peaks (brackets) of E3s. **d** Flotation assay assessing E3 reconstitution in liposomes with varying SFA content. E3s co-migrate with liposomes to top fractions of a 0-40% (w/v) Nycodenz gradient; gradient fractions analyzed by SDS-PAGE and fluorescence scanning. **e** Protease protection assay for E3 orientation. RNF145-TEV-SBP was reconstituted in liposomes with indicated lipid compositions and treated with TEV protease (± 0.8 (w/v) % Triton X-100), followed by SDS–PAGE and anti-SBP western blotting. Numbers below the Western blot images indicate the fraction of signal remaining after the indicated treatments. **f** Purified UBE2G2 analyzed by SDS–PAGE and Coomassie staining; size-exclusion chromatography (HiLoad S75 16/60) with elution peak indicated (bracket). **g** Purified TM_{Cue1}-$AUP1_{271-410}$ analyzed by SDS–PAGE and Coomassie; SEC performed on Superdex 200 10/300 GL. **h** Flotation assay for AUP1 reconstitution in ER-like liposomes. TM_{Cue1}-$AUP1_{271-410}$ co-migrates with liposomes to top fractions of a 0-40% (w/v) Nycodenz gradient; gradient fractions analyzed by SDS-PAGE and stain-free scanning. **i-k** Autoubiquitination of the E3 ligases RNF145 **(i)**, RNF139 **(j)**, and MARCHF6 **(k)** in the presence of indicated E2s and AUP1 (TM_{Cue1}-$AUP1_{271-410}$). Proteins were co-reconstituted in ER-like liposomes (E3/L = 1:8000, TM_{Cue1}-$AUP1_{271-410}$/L = 1:8000, UBE2J2/L = 1:32,000 **(i, j)**; MARCH6/L = 1:8000, TM_{Cue1}-$AUP1_{271-410}$/L = 1:8000, UBE2J2/L = 1:8000 **(k)**; indicated reactions contained 2 μM UBE2G2. **l** Ubiquitin linkage type analysis in reactions with UBE2G2, AUP1 and RNF145 using ubiquitin mutants. ^{DL800}RNF145-ALFA and TM_{Cue1}-$AUP1_{271-410}$ were co-reconstituted in ER-like liposomes (P/L = 1:8000 each). Reactions ± 2 μM UBE2G2 were analyzed after 1 hour by reducing SDS PAGE and fluorescence scanning. **m** Sodium hydroxide (NaOH) treatment to remove oxyester-linked ubiquitin from RNF139 in ER-like liposomes (^{DL800}RNF139-ALFA/L = 1:32,000, UBE2J2/L = 1:32,000). Samples collected at indicated times reduced with DTT, treated with 100 mM NaOH as indicated, and analyzed by SDS PAGE and fluorescence scanning. Detailed liposome compositions are given in Table 1.

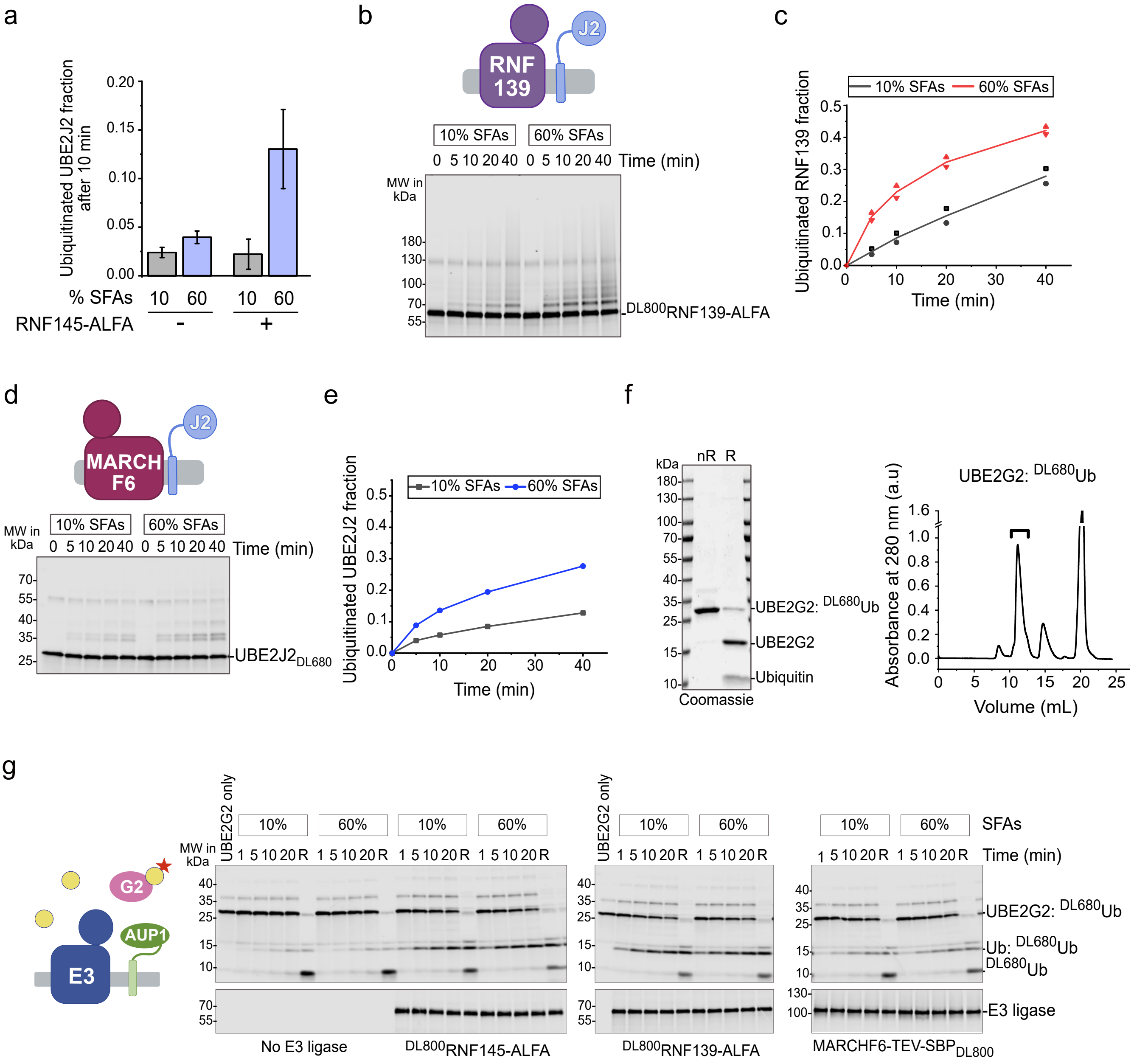

a
Ubiquitinated UBE2J2 fraction after 10 min
0.20
0.15
0.10
0.05
0.00
% SFAs 10 60 10 60
RNF145-ALFA - +
b
RNF 139
J2
10% SFAs
60% SFAs
0 5 10 20 40 0 5 10 20 40 Time (min)
MW in kDa
180
130
100
70
55
DL800RNF139-ALFA
c
10% SFAs
60% SFAs
Ubiquitinated RNF139 fraction
0.5
0.4
0.3
0.2
0.1
0.0
0 10 20 30 40
Time (min)
d
MARCH F6
J2
MW in kDa
10% SFAs
60% SFAs
0 5 10 20 40 0 5 10 20 40 Time (min)
70
55
40
35
25
UBE2J2DL680
e
Ubiquitinated UBE2J2 fraction
10% SFAs
60% SFAs
0.5
0.4
0.3
0.2
0.1
0.0
0 10 20 30 40
Time (min)
f
kDa
nR R
180
130
100
70
55
40
35
25
15
10
UBE2G2: DL680Ub
UBE2G2
Ubiquitin
Coomassie
UBE2G2: DL680Ub
Absorbance at 280 nm (a.u)
1.6
1.0
0.8
0.6
0.4
0.2
0.0
0 5 10 15 20 25
Volume (mL)
g
G2
AUP1
E3
UBE2G2 only
10%
60%
10%
60%
10%
60%
10%
60%
SFAs
MW in kDa
1 5 10 20 R
Time (min)
40
35
25
15
10
70
55
130
100
UBE2G2: DL680Ub
Ub: DL680Ub
DL680Ub
E3 ligase
No E3 ligase
DL800RNF145-ALFA
DL800RNF139-ALFA
MARCHF6-TEV-SBPDL800

Supplementary Figure 7. E3-dependent and E3-independent ubiquitination and E2/E3 discharge assays (related to Fig. 5). a Comparison of E3-dependent and –independent non-cysteine ubiquitination of UBE2J2$_{DL680}$ reconstituted in liposomes with indicated SFA content, with or without RNF145 (P/L = 1:32,000), after 10 minutes (see Fig. 2a and 5a for data). **b** Auto-ubiquitination of RNF139-ALFA with UBE2J2 in co-reconstituted liposomes with varying SFA content (^{DL800}RNF139-ALFA/L = 1:32,000, UBE2J2/L = 1:32,000. **c** Quantification of ubiquitinated RNF139 from **(b)** (n=2). **d** Ubiquitination of UBE2J2 by MARCHF6 in liposomes with the indicated SFA content (MARCH6-TEV-SBP$_{DL800}$/L = 1:32,000, UBE2J2$_{DL680}$/L = 1:32,000). **e** Quantification of ubiquitinated UBE2J2$_{DL680}$ in **(d)**. **f** Purified ubiquitin-loaded UBE2G2 analyzed by non-reducing or reducing SDS–PAGE (nR, R) and Coomassie staining. Size-exclusion chromatography (Superdex 75 Increase 10/300 GL) with elution peak indicated (bracket). **g** E3-mediated discharge of fluorescent ubiquitin from UBE2G2. The indicated E3 ligases were co-reconstituted with AUP1 in liposomes with the indicated SFA content (E3 ligase/L = 1:16,000, TM$_{Cue1}$-AUP1$_{271-410}$/L= 1:16,000). R = reduced. Detailed liposome compositions are given in Table 1.

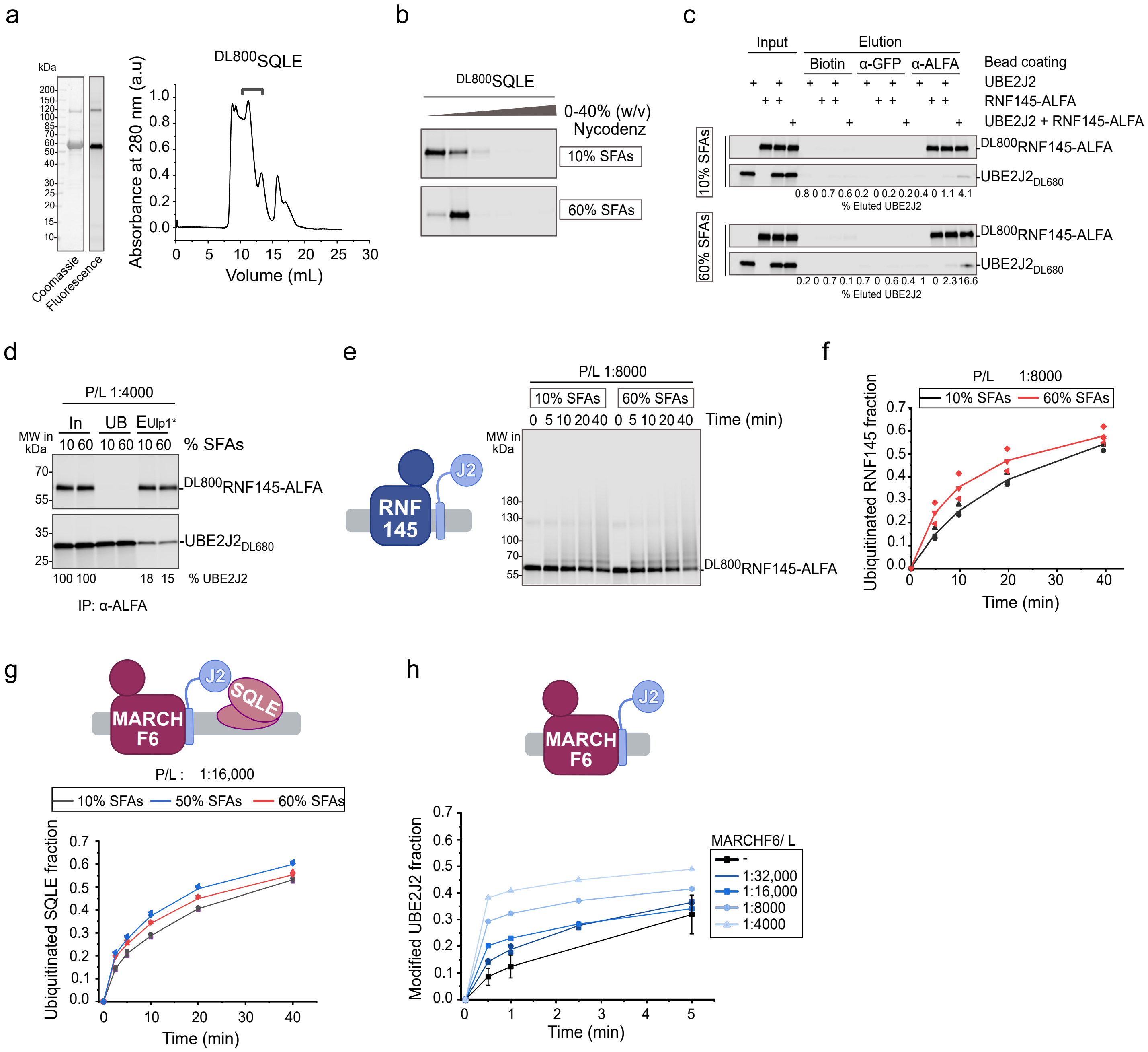
a
kDa
200
150
120
100
85
70
60
50
40
30
25
20
15
10
Coomassie
Fluorescence
DL800SQLE
Absorbance at 280 nm (a.u)
1.0
0.8
0.6
0.4
0.2
0.0
0 5 10 15 20 25 30
Volume (mL)
b
DL800SQLE
0-40% (w/v) Nycodenz
10% SFAs
60% SFAs
c
Input
Elution
Biotin
α-GFP
α-ALFA
Bead coating
UBE2J2
RNF145-ALFA
UBE2J2 + RNF145-ALFA
10% SFAs
60% SFAs
DL800RNF145-ALFA
UBE2J2DL680
0.8 0 0.7 0.6 0.2 0 0.2 0.2 0.4 0 1.1 4.1
% Eluted UBE2J2
0.2 0 0.7 0.1 0.7 0 0.6 0.4 1 0 2.3 16.6
% Eluted UBE2J2
d
P/L 1:4000
In
UB
EUlp1*
MW in kDa
10 60 10 60 10 60
% SFAs
70
55
35
25
DL800RNF145-ALFA
UBE2J2DL680
100 100 18 15
% UBE2J2
IP: α-ALFA
e
RNF 145
J2
P/L 1:8000
10% SFAs
60% SFAs
0 5 10 20 40 0 5 10 20 40
Time (min)
MW in kDa
180
130
100
70
55
DL800RNF145-ALFA
f
P/L 1:8000
10% SFAs
60% SFAs
Ubiquitinated RNF145 fraction
0.7
0.6
0.5
0.4
0.3
0.2
0.1
0.0
0 10 20 30 40
Time (min)
g
MARCH F6
J2
SQLE
P/L : 1:16,000
10% SFAs
50% SFAs
60% SFAs
Ubiquitinated SQLE fraction
0.7
0.6
0.5
0.4
0.3
0.2
0.1
0.0
0 10 20 30 40
Time (min)
h
MARCH F6
J2
Modified UBE2J2 fraction
0.7
0.6
0.5
0.4
0.3
0.2
0.1
0.0
0 1 2 3 4 5
Time (min)
MARCHF6/ L
-
1:32,000
1:16,000
1:8000
1:4000

Supplementary Figure 8. Characterization of SQLE, UBE2J2–E3 interactions, and ubiquitination in reconstituted systems (related to Fig. 5). a Purified SQLE analyzed by SDS-PAGE and Coomassie staining. Size-exclusion chromatography (Superdex 200 10/30 GL) shows SQLE elution peak (bracket). **b** Flotation assay assessing SQLE reconstitution in liposomes with indicated SFA content. SQLE migrates with liposomes to the top fractions of a 0–40% (w/v) Nycodenz gradient; gradient fractions analyzed by SDS–PAGE and fluorescence scanning. **c** Interaction analysis between UBE2J2 and E3 ligase RNF145, reconstituted in the same or separate membranes. ^{DL800}RNF145-ALFA and UBE2J2$_{DL680}$ were individually or co-reconstituted in liposomes (P/L = 1:4000, SFA as indicated), mixed, solubilized, and subjected to immunoprecipitation of RNF145-ALFA using anti-ALFA nanobody. Elution was performed with 2× LDS sample buffer at 50°C or by Ulp1* protease. Proteins were analyzed by SDS–PAGE and fluorescence scanning. Numbers below the gel show fraction of co-purified UBE2J2$_{DL680}$, normalized to input. **d** Analysis of UBE2J2–RNF145-ALFA interaction at higher protein concentrations, by co-reconstitution (P/L = 1:4000, SFA as indicated) and anti-ALFA nanobody immunoprecipitation. Elution by Ulp1* protease; analysis by SDS–PAGE and fluorescence scanning. Numbers below the gel show co-purified UBE2J2$_{DL680}$ normalized to RNF145-ALFA and UBE2J2$_{DL680}$ input. **e** Auto-ubiquitination of RNF145-ALFA with UBE2J2 in liposomes with indicated SFA content at higher P/L ratios (^{DL800}RNF145-ALFA/L = 1:8000, UBE2J2/L = 1:8000). **f** Quantification of reactions in **(e)** (n=3). **g** Quantification of SQLE ubiquitination by MARCHF6 and UBE2J2. ^{DL800}SQLE, UBE2J2, and MARCHF6 were co-reconstituted in PC/PE liposomes with indicated SFA content (P/L = 1:16,000 each). **h** Quantification of UBE2J2$_{DL680}$ ubiquitin loading in the presence of MARCHF6. MARCHF6 co-reconstituted with UBE2J2$_{DL680}$ in liposomes with 10% SFA (UBE2J2$_{DL680}$/L = 1:32,000; MARCHF6/L as indicated). Detailed liposome compositions are given in Table 1.

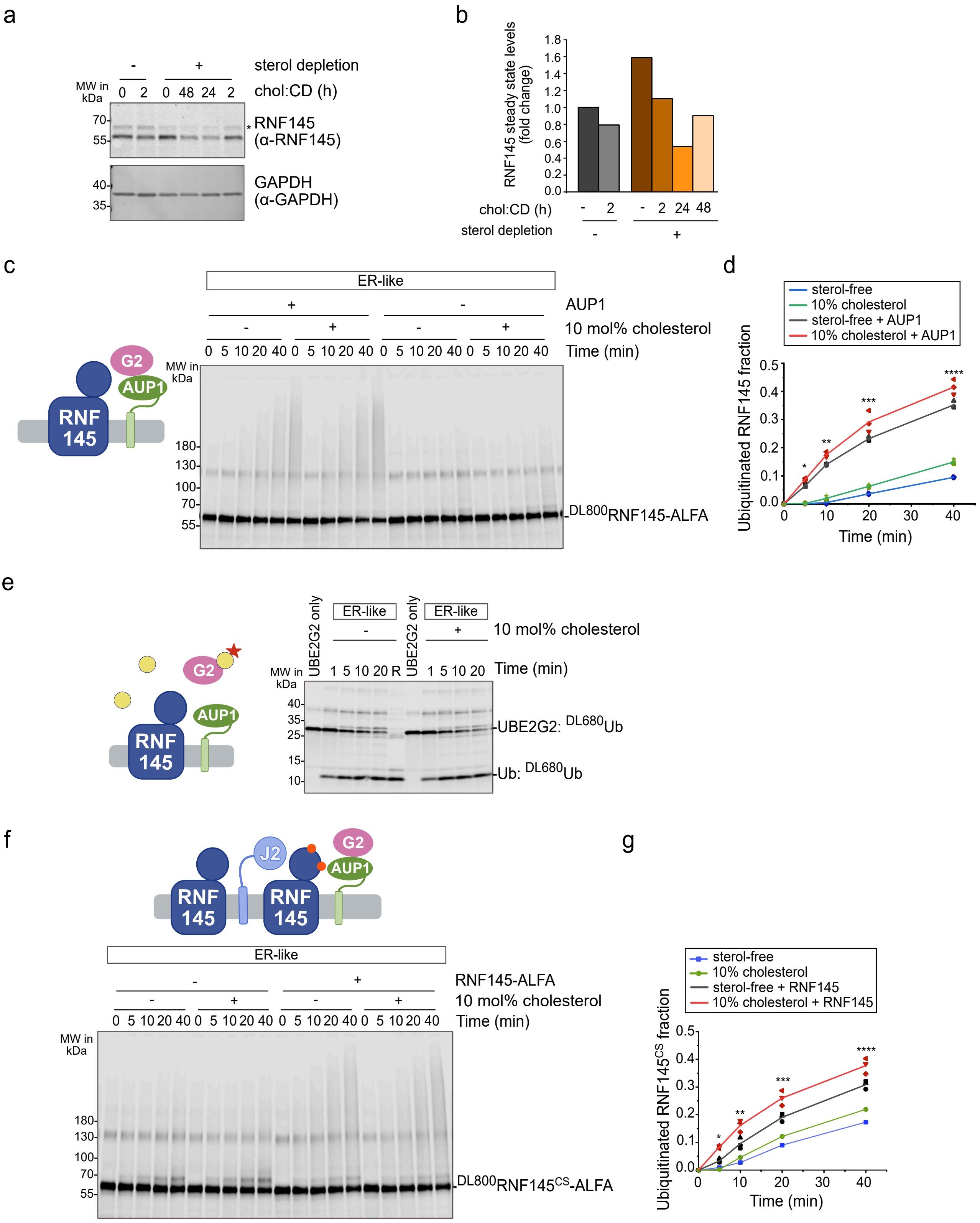
a
sterol depletion
chol:CD (h)
MW in kDa
0 2 0 48 24 2
70
55
RNF145 (α-RNF145)
40
35
GAPDH (α-GAPDH)
b
RNF145 steady state levels (fold change)
1.8 1.6 1.4 1.2 1.0 0.8 0.6 0.4 0.2 0.0
chol:CD (h)
sterol depletion
- 2 - 2 24 48
c
ER-like
AUP1
10 mol% cholesterol
Time (min)
0 5 10 20 40 0 5 10 20 40 0 5 10 20 40 0 5 10 20 40
G2
AUP1
RNF 145
MW in kDa
180
130
100
70
55
DL800RNF145-ALFA
d
sterol-free
10% cholesterol
sterol-free + AUP1
10% cholesterol + AUP1
Ubiquitinated RNF145 fraction
0.5 0.4 0.3 0.2 0.1 0.0
0 10 20 30 40
Time (min)
e
UBE2G2 only
ER-like
10 mol% cholesterol
Time (min)
1 5 10 20 R 1 5 10 20
MW in kDa
40
35
25
15
10
UBE2G2: DL680Ub
Ub: DL680Ub
f
J2
RNF 145
ER-like
RNF145-ALFA
10 mol% cholesterol
Time (min)
0 5 10 20 40 0 5 10 20 40 0 5 10 20 40 0 5 10 20 40
MW in kDa
180
130
100
70
55
DL800RNF145CS-ALFA
g
sterol-free
10% cholesterol
sterol-free + RNF145
10% cholesterol + RNF145
Ubiquitinated RNF145CS fraction
0.5 0.4 0.3 0.2 0.1 0.0
0 10 20 30 40
Time (min)

Supplementary Figure 9. Cholesterol-dependent regulation of RNF145 and related ubiquitination activity (related to Fig. 6). a Steady state levels of RNF145 in HeLa cells under different sterol conditions. After 48 hours of growth under normal (5% FBS) or sterol deplete conditions (5% LPDS), 38 μM cholesterol complexed with β-methyl-cyclodextrin was supplemented in the medium for the indicated amounts of time. Whole cell lysates were analyzed by SDS PAGE and Western blotting. GAPDH served as a loading control. **b** Quantification of RNF145 levels from **(a)**, normalized to GAPDH. **c** Auto-ubiquitination of ^{DL800}RNF145-ALFA with UBE2G2 in ER-like liposomes with or without 10 mol% cholesterol (^{DL800}RNF145-ALFA/L = 1:8000, TM_{Cue1}-AUP1$_{271\text{-}410}$/L = 1:8000). **d** Quantification of reactions in **(c)** (n=3). Statistical significance was assessed by Welch's two-sample t-test at each time point: *p = 0.0089, **p = 0.015, ***p = 0.110, ****p = 0.037. **e** Effect of cholesterol on RNF145-mediated discharge of fluorescent ubiquitin from UBE2G2. RNF145 was co-reconstituted with AUP1 in ER-like liposomes with or without 10 mol% cholesterol (^{DL800}RNF145-ALFA /L = 1:16,000, TM_{Cue1}-AUP1$_{271\text{-}410}$/L = 1:16,000). **f** Ubiquitination of catalytically inactive ^{DL800}RNF145^{CS}-ALFA in the presence of active RNF145 in ER-like liposomes with or without 10 mol% cholesterol (^{DL800}RNF145^{CS}-ALFA/L = 1:8000, RNF145-ALFA/L = 1:8000, UBE2J2/L = 1:32,000, TM_{Cue1}-AUP1$_{271\text{-}410}$/L = 1:32,000). **g** Quantification of ^{DL800}RNF145^{CS}-ALFA ubiquitination in **(f)** (n=3). Statistical significance by Welch's two-sample t-test at each time point: *p = 0.0001, **p = 0.018, ***p = 0.029, ****p = 0.032. Detailed liposome compositions are given in Table 1.

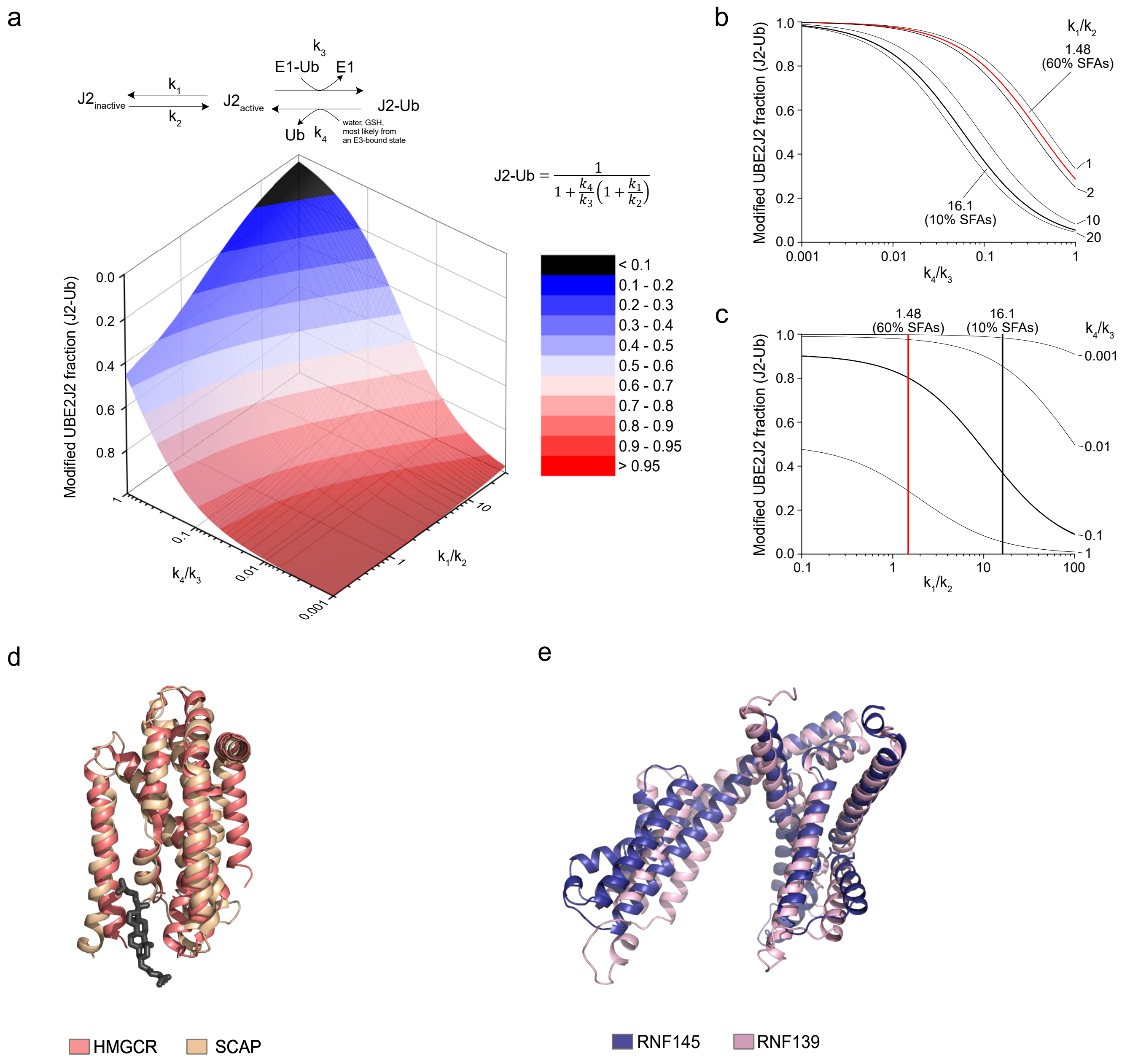

a
b
c
d
e
J2-Ub = 1 / (1 + k4/k3 (1 + k1/k2))
Modified UBE2J2 fraction (J2-Ub)
k4/k3
k1/k2
1.48 (60% SFAs)
16.1 (10% SFAs)
water, GSH, most likely from an E3-bound state
HMGCR
SCAP
RNF145
RNF139

Supplementary Figure 10. Kinetic modelling UBE2J2 Ub-loading, and comparison of sterol sensing domains (SSDs) among SCAP, HMGCR, RNF145, and RNF139 (related to Discussion). a Schematic reaction scheme and kinetic model describing the equilibrium between active and membrane-inactivated UBE2J2 (J2), with rate constants k_1 and k_2; k_3 and k_4 are pseudo-first-order rate constants for ubiquitin loading and discharge reaction, respectively. Lower panel: 3D surface plot showing the steady-state fraction of ubiquitin-loaded UBE2J2 (J2-Ub) as a function of the ratios k_1/k_2 and k_4/k_3, color-coded as indicated. **b** 2D plot depicting the steady-state J2-Ub fraction as a function of k_4/k_3 for selected k_1/k_2 values. Experimentally derived k_1/k_2 values from Fig. 2c, d are shown in bold (black and red lines for membranes with 10% and 60% saturated fatty acids (SFA), respectively). **c** As in (**b**) but plotting J2-Ub fraction as a function of k_1/k_2 for fixed values of k_4/k_3. Vertical lines indicate conditions corresponding to experiments with 10% and 60% SFAs. **d** Structural alignment of the membrane domains of HMGCR (PDB: 8DJM, https://doi.org/10.2210/pdb8DJM/pdb)[3] and SCAP (PDB: 7ETW, https://doi.org/10.2210/pdb7ETW/pdb)[4]. Both exhibit a kink in TM4 that forms a cleft accommodating a sterol molecule (shown as sticks). **e** AlphaFold2-predicted structures of the N-terminal domains of RNF145 (residues 1–307) and RNF139 (residues 1–321) reveal strong similarity to each other, but notable structural divergence from the SSDs of SCAP and HMGCR[5].

Supplementary Table 1 List of lipids for liposome preparation and detergents

Lipids	Full name	Source
DOPC	1,2-dioleoyl-sn-glycero-3-phosphocholine	850375P (Avanti)
POPC	1-palmitoyl-2-oleoyl-glycero-3-phosphocholine	850457P (Avanti)
16:1 (Δ9-Cis) PC	1,2-dipalmitoleoyl-sn-glycero-3-phosphocholine	850358 (Avanti)
DPPC	1,2-dipalmitoyl-sn-glycero-3-phosphocholine	850355P (Avanti)
DOPE	1,2-dioleoyl-sn-glycero-3-phosphoethanolamine	850725P (Avanti)
16:1 PE	1,2-dipalmitoleoyl-sn-glycero-3-phosphoethanolamine	850706 (Avanti)
POPE	1-palmitoyl-2-oleoyl-sn-glycero-3-phosphoethanolamine	850757P (Avanti)
DOPS	1,2-dioleoyl-sn-glycero-3-phospho-L-serine	840035P (Avanti)
Cholesterol	Cholesterol (ovine wool, >98%)	700000P (Avanti)
DM	n-Decyl β-maltoside	Glycon Biochemicals
DDM	n-Dodecyl α-maltoside	Roth
GDN	glyco-diosgenin	Anatrace
DMNG	Decyl Maltose Neopentyl Glycol	Anatrace

OG	n-Octyl β-D-glucopyranoside	Glycon Biochemicals
Triton-X100	Anapoe-X-100	Anatrace

Supplementary Table 2 List of media and supplements for yeast and bacterial cultures

Medium and supplements	Catalogue number	Company
Yeast Nitrogen Base	C19032801	US Biological Life Sciences
Yeast extract	8013-01-2	Roth
Tryptone/ Peptone	91079-40-2	Roth
CSM (-HTLU)	DCS1389	Formedium
CSM (-U)	DCS0161	Formedium
Histidine	DOC0142	Formedium
Leucine	DOC0154	Formedium
Uracil	DOC0214	Formedium
Tryptophan	DOC0186	Formedium
D (+) -Galactose	A1131	PanReac AppliChem
D (+) -Glucose anhydrous	GLU04	Formedium

Supplementary Table 3. List of cell culture media and reagents

Reagent	Catalogue Number	Source
DMEM	D6429	Sigma-Aldrich
Lipoprotein-deficient serum (LPDS)	FB-1001L	Biosera
Trypsin/ EDTA	BE17-161E	Lonza
Penicillin/streptomycin	09-757F	Lonza
FBS	P30-8500	PAN biotech
Mevastatin	M2537	Sigma-Aldrich
Mevalonolactone	M4467	Sigma-Aldrich
β-Methyl-cyclodextrin	C4555	Sigma-Aldrich

Supplementary Table 4 Genes and Uniprot IDs.

Protein	Organism	Protein ID
UBE2J2	*Homo sapiens*	Q8N2K1
UBE2J1	*Homo sapiens*	Q9Y385
RNF145	*Homo sapiens*	Q96MT1
RNF139	*Homo sapiens*	Q8WU17
MARCHF6	*Homo sapiens*	O60337
SQLE	*Homo sapiens*	Q14534
UBE2G2	*Homo sapiens*	P60604
AUP1	*Homo sapiens*	Q9Y679
Uba1	*Saccharomyces cerevisiae*	P22515
Cue1	*Saccharomyces cerevisiae*	P38428
Synaptobrevin 2	*Rattus norvegicus*	P63045

Supplementary Table 5. List of expression constructs

Construct	Source
pET39 His14-SUMO-UBE2J2-LPETGG	This study
pET39 His14-SUMO-UBE2J2^{C94A}-LPETGG	This study
pET39 His14-SUMO-UBE2J2^{C94only}-LPETGG	This study
K27 His14-SUMO-UBE2J2$_{1\text{-}226}$-LPETGG	This study
K27 His14-SUMO-UBE2J2-TM$_{Syb2}$-LPETGG	This study
K27 His14-SUMO-UBE2J2$_{1\text{-}180}$-UBE2J1$_{193\text{-}318}$-LPETGG	This study
K27 His14-SUMO-UBE2J2$_{1\text{-}180}$-UBE2J1$_{193\text{-}287}$-UBE2J2$_{228\text{-}259}$-LPETGG	This study
pET39 His14-SUMO-UBE2J1-LPETGG	This study
pET39 His14-SUMO-UBE2J1$_{1\text{-}192}$-UBE2J2$_{181\text{-}227}$-UBE2J1$_{288\text{-}318}$-LPETGG	This study
pET39 His14-SUMO-UBE2J1$_{1\text{-}192}$-UBE2J2$_{228\text{-}259}$-LPETGG	This study
K27 His14-SUMO-UBE2G2	This study
pET39 His14-SUMO-TM$_{Cue1}$-AUP1$_{271\text{-}410}$	This study
pYTK pGal1-SBP-SUMO$_{EuB}$-GGC-RNF145-ALFA	This study
pYTK pGal1-SBP-SUMO$_{EuB}$-GGC-RNF145$_{C537,540S}$-ALFA	This study
pYTK pGal1-SBP-SUMO$_{EuB}$-GGC-RNF145$_{\Delta RING}$-sfGFP	This study
pYTK pGal1-Avi-SUMO*- linker-RNF145-TEV-SBP	This study
pYTK pGal1-SBP-SUMO$_{EuB}$-GGC-RNF139-ALFA	This study
pYTK MARCHF6-TEV-SBP-LPETGG	This study
pYTK SBP-SUMO$_{EuB}$-MARCHF6	This study
pYTK SBP-SUMO$_{EuB}$-SQLE	This study
K27 untagged yeast ubiquitin	This study
pRS426 pGAL1-His14-TEV-Uba1	[6]
pSF1878 His14-TEV-Ulp1* protease	[7]
pTG-A326 His14-TEV-Ulp1$_{406\text{-}621}$ protease	[6]
pAV286 His14-TEV-SENP$_{EuB}$ protease	[8]
pRK793 MBP-TEVsite-His7- TEV protease	[9]
K27 His-SUMO-Syb2 with C-terminal cysteine (1-117C)	[10]
His14-AVI-SUMO*-αGFP nanobody	[11]
His14-AVI-SUMO*- αALFA nanobody	This study

Supplementary Discussion

Under the experimental conditions employed here for loading assays, UBE2J2 is first allowed to equilibrate between the active and inactive forms before the addition of E1. The subsequent formation of loaded UBE2J2 is effectively irreversible, as discharge occurs only via E3-independent hydrolysis, which is slow. Thus, after extended incubation with E1, nearly all UBE2J2 becomes loaded with ubiquitin regardless of membrane packing, and the steady-state concentration of loaded UBE2J2 is largely unaffected by competition from off-pathway reactions induced by the lipid environment.

In living cells, however, the situation is markedly different. Several factors can greatly accelerate the discharge of ubiquitin from E2 conjugates. First, the presence of E3 ligases increases the rate of hydrolysis by several orders of magnitude. Second, small molecules such as glutathione – abundant in the cytosol at concentrations between 1–10 mM – can further promote discharge. Specifically for UBE2J2, interaction with MARCHF6 strongly enhances the rate of hydrolysis, with hydrolysis rate increasing to values above 0.1 s^{-1} [12], allowing for rapid cycling between loaded and discharged states.

Given the fast E1-mediated loading kinetics as observed here, an important question is whether the relatively slow off-pathway leading to UBE2J2 inactivation can effectively reduce the steady state concentration of ubiquitin-loaded UBE2J2. To address this question, we modeled the pathway by extending the model (Fig. 2d) to include a generic pseudo-first order discharge step (k_4), which can represent hydrolysis or discharge onto a substrate or small molecule, from either an E3-bound or E3-free state. The model also assumes that ubiquitin loading is a pseudo-first order reaction (k_3), as the concentration of loaded E1 should remain constant, although 2 ATP molecules are consumed in every cycle:

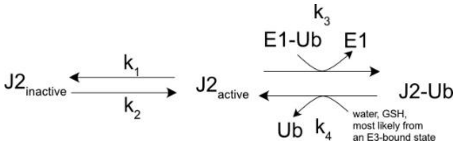

Or, in a more generic form:

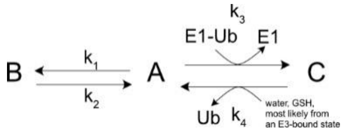

The system can be described by the following set of differential equations:

$$\frac{\mathrm{d}[C]}{\mathrm{d}t} = k_3[A] - k_4[C] \quad (1)$$

$$\frac{\mathrm{d}[A]}{\mathrm{d}t} = -k_3[A] - k_1[A] + k_2[B] + k_4[C] \quad (2)$$

$$\frac{\mathrm{d}[B]}{\mathrm{d}t} = k_1[A] - k_2[B] \quad (3)$$

At steady state:

$$0 = k_3[A] - k_4[C], \text{and } 0 = k_1[A] - k_2[B] \quad (4)$$

resulting in:

$$[C] = \frac{k_3}{k_4}[A], \text{and } [B] = \frac{k_1}{k_2}[A]. \quad (5)$$

Together with $[A]+[B]+[C]=T$, this results in the following expression for the steady-state concentration for $[A]$:

$$[A] = \frac{T}{\left(1+\frac{k_1}{k_2}+\frac{k_3}{k_4}\right)} \quad (6)$$

With $[C] = \frac{k_3}{k_4}[A]$ it follows:

$$[C] = \frac{k_3}{k_4}\frac{T}{\left(1+\frac{k_1}{k_2}+\frac{k_3}{k_4}\right)} \quad (7)$$

$$[C] = \frac{T}{\frac{k_4}{k_3}\left(1+\frac{k_1}{k_2}+\frac{k_3}{k_4}\right)} \quad (8)$$

$$[C] = \frac{T}{\left(\frac{k_4}{k_3}+\frac{k_1k_4}{k_2k_3}+1\right)} \quad (9)$$

After some rearrangement, this yields the equation plotted in Supplementary Fig. 10a-c:

$$[C] = \frac{T}{1+\frac{k_4}{k_3}\left(1+\frac{k_1}{k_2}\right)} \quad (10)$$

When discharge is much slower than loading ($k_4 \ll k_3$), the denominator remains close to one, resulting in a high proportion of loaded enzyme that is largely insensitive to the equilibrium between active and inactivated forms. Under these conditions, the membrane-interaction off-pathway, governed by k_1/k_2, has minimal regulatory impact. However, as the discharge rate increases, the relative steady-state population of loaded UBE2J2 becomes progressively sensitive to the equilibrium between active and inactive states. In this situation, frequent transitions between loaded and unmodified forms expose UBE2J2 to repeated opportunities for inactivation via the membrane-dependent pathway. Consequently, the lipid environment, by modulating k_1/k_2, can exert a substantial influence on the overall fraction of functionally loaded enzyme. Thus, the capacity of the off-pathway to modulate ERAD efficiency critically depends on the dynamic cycling between loading and discharge.

Supplementary References

1. Ballweg, S., Sezgin, E., Doktorova, M., Covino, R., Reinhard, J., Wunnicke, D., Hänelt, I., Levental, I., Hummer, G. & Ernst, R. Regulation of lipid saturation without sensing membrane fluidity. *Nature communications* **11**, 756 (2020). https://doi.org:10.1038/s41467-020-14528-1
2. Gautier, R., Bacle, A., Tiberti, M. L., Fuchs, P. F., Vanni, S. & Antonny, B. PackMem: A Versatile Tool to Compute and Visualize Interfacial Packing Defects in Lipid Bilayers. *Biophysical journal* **115**, 436-444 (2018). https://doi.org:10.1016/j.bpj.2018.06.025
3. Chen, H., Qi, X., Faulkner, R. A., Schumacher, M. M., Donnelly, L. M., DeBose-Boyd, R. A. & Li, X. Regulated degradation of HMG CoA reductase requires conformational changes in sterol-sensing domain. *Nature communications* **13**, 4273 (2022). https://doi.org:10.1038/s41467-022-32025-5
4. Yan, R., Cao, P., Song, W., Qian, H., Du, X., Coates, H. W., Zhao, X., Li, Y., Gao, S., Gong, X., Liu, X., Sui, J., Lei, J., Yang, H., Brown, A. J., Zhou, Q., Yan, C. & Yan, N. A structure of human Scap bound to Insig-

2 suggests how their interaction is regulated by sterols. *Science* **371** (2021). https://doi.org:10.1126/science.abb2224
5. Jumper, J., Evans, R., Pritzel, A., Green, T., Figurnov, M., Ronneberger, O., Tunyasuvunakool, K., Bates, R., Zidek, A., Potapenko, A., Bridgland, A., Meyer, C., Kohl, S. A. A., Ballard, A. J., Cowie, A., Romera-Paredes, B., Nikolov, S., Jain, R., Adler, J., Back, T., Petersen, S., Reiman, D., Clancy, E., Zielinski, M., Steinegger, M., Pacholska, M., Berghammer, T., Bodenstein, S., Silver, D., Vinyals, O., Senior, A. W., Kavukcuoglu, K., Kohli, P. & Hassabis, D. Highly accurate protein structure prediction with AlphaFold. *Nature* **596**, 583-589 (2021). https://doi.org:10.1038/s41586-021-03819-2
6. Stein, A., Ruggiano, A., Carvalho, P. & Rapoport, T. A. Key steps in ERAD of luminal ER proteins reconstituted with purified components. *Cell* **158**, 1375-1388 (2014). https://doi.org:10.1016/j.cell.2014.07.050
7. Frey, S. & Görlich, D. A new set of highly efficient, tag-cleaving proteases for purifying recombinant proteins. *Journal of chromatography. A* **1337**, 95-105 (2014). https://doi.org:10.1016/j.chroma.2014.02.029
8. Vera Rodriguez, A., Frey, S. & Gorlich, D. Engineered SUMO/protease system identifies Pdr6 as a bidirectional nuclear transport receptor. *J Cell Biol* **218**, 2006-2020 (2019). https://doi.org:10.1083/jcb.201812091
9. Kapust, R. B., Tozser, J., Fox, J. D., Anderson, D. E., Cherry, S., Copeland, T. D. & Waugh, D. S. Tobacco etch virus protease: mechanism of autolysis and rational design of stable mutants with wild-type catalytic proficiency. *Protein Eng* **14**, 993-1000 (2001). https://doi.org:10.1093/protein/14.12.993
10. Schmidt, C. C., Vasic, V. & Stein, A. Doa10 is a membrane protein retrotranslocase in ER-associated protein degradation. *Elife* **9** (2020). https://doi.org:10.7554/eLife.56945
11. Pleiner, T., Bates, M., Trakhanov, S., Lee, C.-T., Schliep, J. E., Chug, H., Böhning, M., Stark, H., Urlaub, H. & Görlich, D. Nanobodies: site-specific labeling for super-resolution imaging, rapid epitope-mapping and native protein complex isolation. *eLife* **4**, e11349 (2015). https://doi.org:10.7554/eLife.11349
12. Swarnkar, A., Leidner, F., Rout, A. K., Ainatzi, S., Schmidt, C. C., Becker, S., Urlaub, H., Griesinger, C., Grubmuller, H. & Stein, A. Determinants of chemoselectivity in ubiquitination by the J2 family of ubiquitin-conjugating enzymes. *EMBO J* **43**, 6705-6739 (2024). https://doi.org:10.1038/s44318-024-00301-3
